# Contributing factors for urban-rural inequalities in unmet need for family planning among reproductive-aged women in Ethiopia: a Blinder-Oaxaca decomposition analysis

**DOI:** 10.1186/s12905-023-02304-4

**Published:** 2023-04-04

**Authors:** Henok Mulatu Teferi, Julia Schröders

**Affiliations:** 1 Umeå International School of Public Health, Umeå University, Umeå, Sweden; 2 Department of Epidemiology and Global Health, Umeå University, Umeå, SE-901 87 Sweden

**Keywords:** Urban-rural inequalities, Reproductive health, Unmet FP needs, Health inequalities, Maternal health

## Abstract

**Background:**

Unmet need for family planning (FP) is a global public health concern, particularly in low- and middle-income countries. In Ethiopia, although several studies have assessed unmet needs for FP, there have only been few empirical investigations into regional inequalities and their contributory factors. This study assessed urban-rural inequalities in unmet FP needs among reproductive-aged women in Ethiopia and particularly examined the contribution of material, cultural-behavioral, and psychosocial factors therein.

**Methods:**

A cross sectional study was conducted among 8811 reproductive-aged women derived from the nationally representative 2019 Ethiopian Performance Monitoring for Action (PMA) data. The outcome variable was unmet need for FP. The exposure variable was place of residence (urban or rural). Contributing factors were categorized into material, psychosocial and cultural-behavioral factors. Blinder-Oaxaca decomposition analysis was used to assess urban-rural inequalities in unmet need for FP as well as to disentangle the contributory factors in percentage points.

**Result:**

In our study, 13.8% of reproductive-aged women in Ethiopia reported unmet FP needs. Urban-rural inequalities therein accounted for 6.8% points. Disparities in FP needs between urban and rural areas were mostly explained by psychosocial factors (81.0%) followed by material (21.0%), and cultural-behavioral (3.2%) factors. While women who were living with a partner (39.1%, p < 0.01) and multiparas (51%, p < 0.01) contributed to increasing inequalities, attending family planning counseling services with a healthcare provider (-1.7%, p = 0.03) reduced the gap in unmet need for FP between urban and rural areas. Women from the poorest and poor category contributed 14.1% (p = 0.02) and 11.1% (p = 0.04), respectively. Being from a Muslim religion also contributed to the disparity by 7.3% (p < 0.01).

**Conclusion:**

This study showed that among reproductive-aged women in Ethiopia, inequalities in unmet FP needs show distinct urban-rural patterning. Most inequalities could be attributed to psychosocial factors, mainly parity and marital status, followed by material and cultural-behavioral factors. Policymakers should target these modifiable psychosocial factors to reduce urban-rural inequalities in unmet need for FP in Ethiopia.

## Background

Unmet need for family planning (FP) is a global public health concern, particularly in low- and middle-income countries (LMICs). The World Health Organization (WHO) defines unmet need for FP as those sexually active and fecund women who are not using any type of contraceptive despite not wanting to have more children (= unmet need for limiting) or wanting to delay the next child (= unmet need for spacing) [1]. As asserted in the Agenda 2030, unmet need for FP is one of the indicators used for measuring improvements in access to reproductive health services [2] and the overall progress towards Sustainable Development Goal (SDG) 3 aiming to promote good health and wellbeing.

While globally, approximately one out of every ten women has an unmet need for FP, the majority of unmet needs occurs among women in LMICs [2]. According to a study conducted in sub-Saharan Africa, the prevalence of unmet need for FP was 27% [3], with Ethiopia being one of the countries with the highest prevalence. A systematic review and meta-analysis of 15 studies published between 2005 and 2018 involving 17,585 reproductive-aged women revealed that between 26.5% and 36.4% of Ethiopian reproductive-aged women have an unmet need for FP, which is significantly higher than the prevalence in other Sub-Saharan African countries [4].

Previous studies show that 74% of unwanted pregnancies in LMICs, and 86% in East Africa, are due to an unmet need for family planning services [5–8]. Inequalities in unmet needs for FP have been identified across several countries. Prior studies identified inequalities in unmet need for FP as a result of differences in various factors including marital status, parity, women’s education, husbands’ education, household wealth, and access to FP services [9–14]. It has been shown that women who are married, and multiparas more often face unmet needs for FP as compared to those who are single and nulliparas, respectively [10, 14]. Low-educated women and women living with a husband with low-educational attainment also had higher risks of unmet need for FP compared to their more educated counterparts, respectively [11, 13, 14]. Similarly, unmet FP needs are more prevalent among women with low socio-economic status and those with poor access to FP services [9, 10, 12–17].

Various studies have shown urban-rural differences in unmet need for FP among reproductive-aged women [13, 15, 17–19]. Prior studies conducted in Bangladesh and Pakistan have shown that married women who reside in rural areas have more unmet needs for FP as compared to those who reside in urban areas [13, 20]. A study conducted in a number of sub-Saharan African countries has also shown urban-rural inequalities in unmet need for FP [21]. Similar results have been published for the Ethiopian context [15, 17, 18]. For example, a nationally representative study conducted in Ethiopia has shown that the prevalence of unmet need for FP in rural areas was higher than in urban areas (19.1% vs. 7.2%) [15].

Even though prior studies have identified urban-rural inequalities in unmet need for FP, including for the Ethiopian context, there is a limited number of studies that assessed the contributory factors for these inequalities [13, 22, 23]. Understanding the contributory factors for such urban-rural disparities in unmet need for FP is important for guiding policy and practice in reducing unmet FP needs among Ethiopian women.

This study therefore set out to assess urban-rural inequalities in unmet FP needs among reproductive-aged women in Ethiopia and to explore the contribution of material, cultural-behavioral, and psychosocial factors to the observed inequalities.

## Methods

### Study design

This is a cross-sectional study using the 2019 Ethiopian Performance Monitoring for Action (PMA) survey. The data is publicly available and can be accessed upon request from the PMA website (https://www.pmadata.org/countries/ethiopia). The PMA is a nationally representative data collected on key reproductive, maternal, and child health indicators by Addis Ababa University in collaboration with Johns Hopkins Bloomberg School of Public Health and the Ethiopian Ministry of Health [24].

### Sampling, study population, and data collection

PMA Ethiopia employed a two-stage cluster sampling using the place of residence and regions as sampling stratum. Using the master sample frame from the Ethiopian Central Statistical Agency (CSA), a total of 265 enumeration areas (EAs) were selected [24]. Then, from each EA, 35 households were randomly sampled. Finally, 9254 households were selected to participate in the survey and 9108 households consented to and completed the survey [24]. All reproductive aged women between the ages of 15 to 49 years and who are members of the household or who stayed in the household the night before the day of the interview were eligible to complete the female questionaries for the PMA survey. A total of 8975 eligible women were identified. Out of the identified women, 8837 (98.5%) completed the female questionnaire and those were the target population in this study [24]. Among 8837 women who completed the survey, 26 had incomplete responses and were subsequently dropped. Finally, a total of 8811 women, which accounted 99.7% of the total women who completed the female questionnaire of the PMA survey sample were included in the present analysis. The sample size of the Ethiopian PMA survey was estimated using the Wilson method which is based on a recently available modern contraceptive prevalence rate from prior studies assuming a 5% margin of error [25]. The data was collected from September to December 2019 by well-trained resident enumerators [24].

### Measurements

#### Outcome variable

*Unmet need for family planning*: this was a binary variable and readily available in the PMA Ethiopia data. It was measured by a sequence of steps: first, all women of reproductive age, i.e., those between the age of 15–49 years were asked whether they are using any method of contraception, or not. Second, those who responded that they were not using any contraception were further asked whether they are currently pregnant/postpartum amenorrheic, or not pregnant. Those who were currently pregnant / postpartum amenorrheic but did not want the current pregnancy/last pregnancy at all (= unmet need for limiting) or wanted the current pregnancy later (= unmet need for spacing) were considered as “*unmet need for FP.”* Similarly, fecund women who were not pregnant or postpartum amenorrheic and were not using contraceptives while they do not wish to become pregnant at all (= unmet need for limiting) and within the next two years wanted a child but were undecided on the timing, and/or undecided on wanting a child (= unmet need for spacing) were also categorized as “*unmet need for FP*.” The rest were grouped under “*no unmet need for FP*.” The steps for calculating this variable is available on the PMA data analysis handbook.

#### Exposure variable

*Place of residence* was the exposure variable and was coded as “0” (rural) and “1” (urban).

#### Explanatory variables

In order to identify and classify relevant contributing factors to the disparities in unmet need for FP across urban and rural areas, we used the explanatory pathways for health inequalities conceptual framework described by Ravindran and Gaitonde [26]. Five distinct paths for health inequalities were described by the framework, including the materialist, cultural-behavioural, psychosocial, neo-materialist, and life-cycle based pathways [26]. Neo-materialist and life-cycle based paths were excluded due to data limitations.

##### Material variables

The material explanation contends that those with lower socioeconomic status (SEP) lack the material and economic means to gain access to resources and goods critical to health, such as healthcare services [26]. This pathway explains how individual health is impacted by various types of impoverishments [26]. Variables under this category included: *Level of education* (‘no education’, ‘primary education’, and ‘secondary or higher’), *wealth quintile* (poorest, poor, middle, rich, richest), and *region* (‘emerging region’: these are regions that share a common characteristics of harsh weather conditions and socioeconomically disadvantage compared to the other regions and includes: Afar, Somali, Benishangul-Gumz and Gambela’ [27], ‘developed region’: these are regions performing better in every sector compared to the emerging regions and includes: Amhara, Oromia, SNNPs region, Harari and Tigray’, and ‘city administration’: Addis Ababa and Dire Dawa’).

##### Cultural-behavioral variables

This pathway illustrates how various cultural ideas, norms, and practices in the community, as well as individual behaviors can have an impact on individual health and lead to health disparities [26]. The included variables were: *religion* (‘Christians’: Orthodox, Protestant and Catholics, ‘Muslim’ and ‘other religion’: Wakefeta, traditional, other and no religion*)*, *media exposure* (*‘*have media exposure for FP’ and ‘have no media exposure for FP’). Media exposure is included as a behavioral component because women’s individual behaviors in utilizing media, such as listening to the radio, reading magazines, and using social media, may vary.

##### Psychosocial variables

The psychosocial explanation emphasizes the role of social networks to provide opportunities for community and social support [26]. The literature further argues that health inequalities result from differential experiences in psychosocial distress and psychosocial support among groups with different SEP. Variables under this category included: *marital status* (dichotomized into “single” *(i.e.*, never married, divorced, and/or widowed) and “living with a partner” which includes women who are either married and live with a partner or those who are not married but live with their partners); *attended FP counseling services by a healthcare provider* (dichotomized into ‘yes’ and ‘no’), *parity* (‘nullipara: women who have no children’, ‘primipara: those who have only one child’, and ‘multipara: those who have given birth twice or more’). Attending a healthcare provider may provide women with psychosocial support by allowing them to talk about any potential side effects and worries they may have with the use of FP. The degree of social support may also vary based on parity, where primiparas have better support than multiparas [28]. To capture “*perceived community support”*, women were asked three questions regarding their community norms towards facility delivery, antenatal care, and postnatal care. The questions were, “Do most, some, few, or no people in your community encourage women to deliver at a facility?”, “Do most, some, few, or no people in your community encourage women to go to Antenatal care (ANC)?”, and “Do most, some, few or no people in your community encourage women to go to postnatal care?”, respectively. Based on these questions, those women who responded: “most people” in either of the three questions were coded as “0” (high community support), and those who responded to the rest were coded as “1” (low community support).

#### Covariates

##### Maternal age

maternal age was treated as a covariate and categorized into three categories (“15–24”, “25–34”, and ’35–49’).

### Statistical analysis

Both descriptive and analytical statistics were used in this study. First, description of the overall characteristics of the study participants as well as cross-tabulating the explanatory variables with the exposure ‘place of residence’ were determined and presented in frequencies and percentages. Pearson’s chi-square was used to assess statistical differences between urban and rural residents among the study participants. Then, a Blinder-Oaxaca decomposition analysis was applied to assess urban-rural inequalities in unmet need for FP among reproductive-aged women in Ethiopia and to determine the contribution of material, cultural-behavioral as well as psychosocial variables to these inequalities.

We used Blinder-Oaxaca decomposition analysis because of its use to explain the difference in the mean of an outcome variable between two groups and decompose it to determine the contributions of the explanatory variables to this difference [29]. The analysis results in an explained part by the observed characteristics and an unexplained part. Then, the individual explanatory variables (material, cultural-behavioral, and psychosocial variables) contributions to the explained differences in unmet need for FP between women who live in urban areas and those who live in rural areas were estimated. The variance inflation factor (VIF) was also assessed to determine the multicollinearity among the included explanatory variables and all the explanatory variables have a VIF value of less than 3 [30]. Based on the PMA data analysis manual, weighting was applied to adjust for selection probability and non-response. All statistical analyses were conducted using Stata 17.0.

The Blinder-Oaxaca decomposition analysis equation was generated from extending the linear regression models. Given the two groups (*urban and rural)*, an outcome variable (*unmet need for FP)*, and a set of *explanatory variables*, the difference in the mean of unmet need for FP between the urban and rural will be:

1 $$R = E({Y_A}) - E({Y_B})$$

Where E(Y) is the expected value of the unmet need for FP and accounted for the urban (A) and rural (B) difference.

Based on the linear model,

$$y = {\beta _{0(t)}} + {\beta _t}{x_t} + {e_t}\,\,\,\,\,E({e_t}) = 0\,\,\,\,\,t = (A\,\,B)$$

Where X is the vector containing the predictor and the constant, $\beta$ contains the slope parameter and the intercept, and e is the error. Then, the mean difference in the unmet need for FP can be expressed as

2 $$\begin{array}{c}R = {Y_{(A)}} - {Y_{(B)}} = ({\beta _{0(A)}} - {\beta _{0(B)}})\\{\mkern 1mu} + {\mkern 1mu} ({\beta _A}{x_A} - {\beta _B}{x_B})\end{array}$$

The difference in y between the urban and the rural can be attributed to differences in the intercepts (${\beta }_{0}$), and differences in “x” and ${\prime }\beta$’.

Then, based on Neumark’s suggestion [29], using the pooled coefficients (${\beta }^{p}$) from the pooled data regression, the equation for the contribution of the urban-rural residence in the explanatory variables for the unmet need for FP will be

3 $$\begin{array}{c}R = \left[ {\{ E({x_A}) - E({X_B})\} *{\beta ^p}} \right]\\\,\,\, + \left[ {E({X_A})*({\beta _A} - {\beta ^p})} \right]\\\,\, + \left[ {E({x_B})*({\beta ^p} - {\beta _B})} \right]\end{array}$$

In the above equation, the explained urban-rural disparities in unmet need for FP by the included explanatory variables are explained by the first part “[{E(X_A_) – E(X_B_)} *ß^p^]”. Whereas, the second part, “[E(X_A_)*(ß_A −_ß^p^)] + [E(X_B_) *(ß^p^ -ß_B_)]”, shows the unexplained part, that is the urban-rural difference in the unobserved part. The updated Oaxaca decomposition package using “logit” was used to conduct the decomposition analysis. The “logit” command computes the non-linear decomposition for a binary outcome using the weighting approach described by Yun [31]. A detailed information on the Blinder-Oaxaca decomposition derivative equation method and its implementation in statical softwares can be found elsewhere [29, 31, 32].

### Ethical approval

PMA Ethiopia received ethical approval both from the Johns Hopkins University Bloomberg School of Public Health institutional review board and Addis Ababa University, College of Health Sciences. Informed oral consent was obtained from the study participants [25].

## Results

### Overall characteristics of the study participants

The overall prevalence of unmet need in FP among reproductive aged women in Ethiopia was 13.8%. In addition, the prevalence of unmet FP needs in Ethiopia is higher in the rural (16%) than the urban (9%) areas. Table 1 shows the overall characteristics of the study participants as well as by place of residence. Among 8811 women included in the study, 40% and 32% were between the age of 15–24 and 25–34 years, respectively compared to those between the ages of 35–49 years (28%). Higher percentage of women between the ages of 35–49 years old were from urban areas (76%) compared to those from the rural areas (24%). Majority of women received no (38%) or only primary education (37%) compared to those who completed secondary or higher education (25%). Secondary or higher education was more common among urban compared to rural women (66% vs. 34%). Moreover, 95% of women in the richest quintile and 99% from the poorest quintile resided in urban and rural areas, respectively. Majority of the study participants were from the developed region (87%) and women who resided in rural areas were more common in the emerging region (64%) compared to the city administration (3%). Among Muslim women included in the study, 77% and 33% of women were from the rural and urban areas, respectively. A high percentage of rural women were single (56%) and primiparas (58%) compared to those who resided in urban areas. While only 8% of women attended FP counseling services with a healthcare provider, majority (71%) had a high perceived community support.

**Table 1:** Weighted frequency and percentages of the overall study participants as well as by place of residence (n = 8811)

Variables	Total	Urban	Rural	P value
	n (%)	n (%)	n (%)	
Total	8811 (100)	2887 (33)	5924 (67)	
**Covariates**				
Age				< 0.001
35–49	2498 (28)	599 (24)	1899 (76)	
25–34	2802 (32)	1017 (36)	1785 (64)	
15–24	3511 (40)	1271 (36)	2240 (64)	
**Material factors**				
Education				< 0.001
No education	3321(38%)	444 (13)	2877 (87)	
Primary education	3234 (37%)	960 (30)	2274 (70)	
Secondary education or higher	2256 (25%)	1483 (66)	773 (34)	
Wealth index				< 0.001
5th quintile	2003 (22.7)	1896 (95)	107 (5)	
4th quintile	1712 (19.4)	760 (44)	952 (56)	
3rd quintile	1708 (19.3)	134 (8)	1574 (92)	
2nd quintile	1697 (19.2)	73 (4)	1624 (96)	
1st quintile	1691 (19.1)	24 (1)	1667 (99)	
Region				< 0.001
Developed region	7699 (87)	2125 (28)	5574 (72)	
Emerging region	525 (6)	190 (36)	335 (64)	
City administration	587 (7)	572 (97)	15 (3)	
**Cultural-behavioral factors**				
Religion				0.03
Christian	6209 (70)	2286 (37)	3923 (63)	
Muslim	2478 (28)	563 (23)	1915 (77)	
Other	124 (2)	38 (30)	86 (70)	
Media exposure				< 0.001
Yes	3488 (40)	1764 (51)	1724 (49)	
No	5323 (60)	1123 (21)	4200 (79)	
**Psychosocial factors**				
Marital status				< 0.001
Single	3015 (34)	1317 (44)	1698 (56)	
Living with a partner	5796 (66)	1570 (27)	4226 (73)	
Parity				< 0.001
Nullipara	2875 (33)	1261 (44)	1614 (56)	
Primipara	2306 (26)	971 (42)	1335 (58)	
Multipara	3630 (41)	655 (18)	2975 (82)	
Attended FP counseling with HCP				< 0.001
No	8071 (92)	2728 (34)	5343 (66)	
Yes	740 (8)	159 (21)	581 (79)	
Perceived Community support				< 0.001
High	6299 (71)	2524 (40)	3775 (60)	
Low	2512 (29)	363(14)	2149 (86)	

### Blinder-Oaxaca decomposition

As displayed in Table 2, urban-rural inequality in unmet need for FP among reproductive-aged women in Ethiopia was 6.864% points with a remaining difference of -0.002% unexplained.

**Fig. 1:**
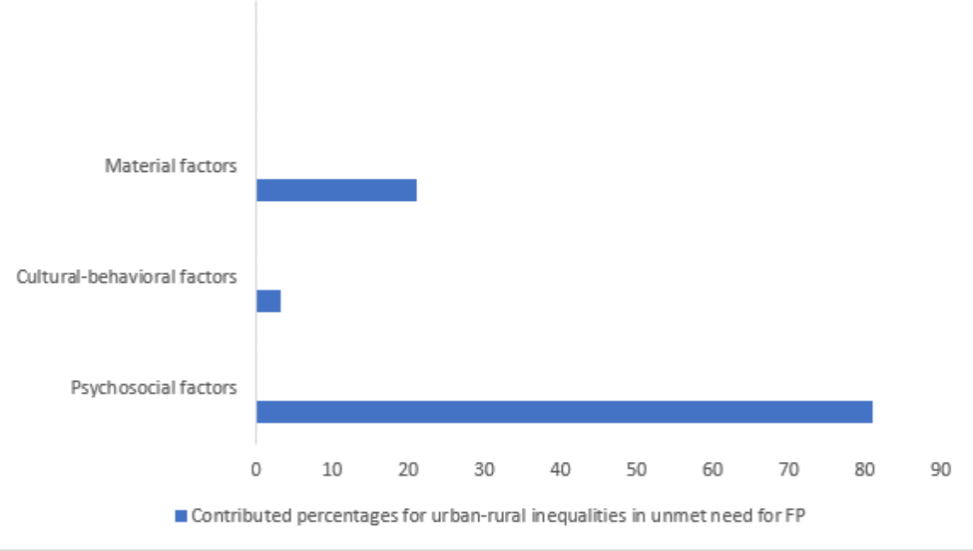
Percentage contribution of the explanatory variables for urban-rural inequalities in unmet need for FP

According to this study, the psychosocial factors were the highest contributor (81%) to urban-rural inequalities in unmet need for FP followed by the material (21%) and cultural-behavioral factors (3%) (Fig. 1). Maternal age reduced the disparity in unmet need for FP between rural and urban areas.

Among the material factors, household wealth has the highest and statistically significant contribution to urban-rural inequalities in unmet need for FP. Being in the poorest and poor group contributed 14.1% (p = 0.02) and 11.1% (p = 0.04) for urban-rural inequalities compared to the richest quintile, respectively. This is because a higher percentage of women with unmet FP needs in rural areas were in the poorest and poor group compared to women with unmet FP needs in the urban areas.

Religion was the statistically significant contributor among the cultural-behavioral factors. It was shown that being Muslim contributed 7.3% (p < 0.01) to the urban-rural inequalities compared to being Christian. This shows that percentages of Muslims were higher among women with unmet need for FP in rural areas than the urban areas. From the psychosocial factors, marital status and parity were the main contributors to the inequalities in the unmet need for FP between the urban and rural areas. Living with a partner increased inequalities by 39.1% (p < 0.01) as compared to being single. Likewise, being multipara contributed 51% (p < 0.01) to the urban-rural inequalities in unmet need for FP compared to the nulliparas. On the other hand, being primipara (-12.2%, p < 0.01) and attending group FP counseling services (-1.7%, p = 0.03) reduced the inequalities.

Maternal age reduced the gap in unmet need for FP between the rural and urban areas. Being between the age of 15–24 years (-1.6%, p = 0.03) and 25–34 years (-3.49%, p = 0.01) reduced the gap between the two groups compared to those between 35 and 49 years of age. The negative result showed that, majority of women among those with unmet FP needs in urban areas were between the age of 15–24 and 25–34 years compared to those with unmet FP need in rural areas.

**Table 2:** Weighted decomposition for urban-rural inequalities in unmet need for family planning among reproductive-aged women in Ethiopia

Unmet need for family planning in Rural		16.1%	
Unmet need for family planning in Urban		9.2%	
Rural-Urban differences		6.864% points	
**Total explained differences**		**6.866% points**	
**Unexplained differences**		**− 0.002% points**	
**Explanatory variables**	**Coefficients**	**% Contribution to the explained difference**	**p-value**
**Covariates**			
Age			
35–49	ref		
25–34	-0.0024	**-3.49**	**0.01**
15–24	-0.0011	**-1.60**	**0.03**
**Material factors**			
Level of education			
No education	ref		
Primary education	-0.0008	-1.2	0.10
Secondary and higher education	0.0012	1.9	0.79
Wealth index			
5th quintile	ref		
4th quintile	-0.002	-3.1	0.12
3rd quintile	0.0026	3.8	0.45
2nd quintile	0.0076	**11.1**	**0.04**
1st quintile	0.0097	**14.1**	**0.02**
Region			
Developed region	ref		
Emerging region	0.0003	0.5	0.18
City administrations	-0.0042	-6.1	0.11
**Cultural and behavioral factors**			
Religion			
Christian	ref		
Muslim	0.005	**7.3**	**< 0.01**
Other	-0.000032	-0.05	0.69
Media exposure			
Have media exposure	ref		
Have no media exposure	-0.002	-4.08	0.27
**Psychosocial factors**			
Marital status			
Single	ref		
Living with a partner	0.026	**39.1**	**< 0.01**
Parity			
Nullipara	ref		
Primipara	-0.008	**-12.2**	**< 0.01**
Multipara	0.035	**51.0**	**< 0.01**
Attended FP counseling services by a healthcare provider			
No	ref		
Yes	-0.0012	**-1.7**	**0.03**
Perceived community support			
Yes	ref		
No	0.0033	4.8	0.09

## Discussion

This study examined unmet needs for FP among reproductive-aged women in Ethiopia and particularly assessed the contribution of material, cultural-behavioral, and psychosocial factors on urban-rural inequalities in unmet FP needs. The results showed that inequalities in unmet needs for FP between urban and rural areas were mostly explained by psychosocial factors, followed by material and cultural-behavioral factors. Like prior studies [18, 20, 21], this study further confirmed the persistence of urban-rural inequalities in unmet need for FP among reproductive-aged women in Ethiopia, which is to the advantage of women who reside in urban areas. However, unlike prior studies, this study also examined the contributing factors from the perspectives of material, cultural-behavioral, and psychosocial explanatory pathways.

The present study showed that psychosocial factors, including parity and marital status, had the highest contribution to urban-rural inequalities in unmet needs for FP. Multiparas contributed most of the inequalities in the unmet need for FP between the urban and rural areas as compared to nulliparas. These findings are consistent with a study conducted in Ethiopia and Burundi, where multiparas were associated with a high prevalence of unmet need for FP [11, 12, 14]. Reasons for such disparities may be due to poor empowerment and lower decision-making power of multipara women in rural areas regarding their FP choice as a result of sociocultural influences and norms prevailing in these areas [12]. Conversely, primiparas had a reduced gap in unmet need for FP between urban and rural areas in this study. Prior studies have identified primiparas to have more social and community support than that of the multipara women [28]. [33].

This study also identified that living with a partner increased inequalities in unmet need for FP for women in urban vs. rural areas. This finding is likewise supported by a prior study conducted in Ethiopia, where married women showed a higher prevalence of unmet FP needs than non-married ones [10, 34]. Studies have also shown that urban non-single women may hold better decision-making powers regarding modern contraceptive use than those women who live in rural areas [18]. In addition, women from the urban areas who live with their partner may have a high chance of getting partner support regarding the issues of sexual and reproductive health than those who live in the rural areas [35]. Financial independence among urban women due to a higher chance of education and job compared to rural women may also contribute to the urban-rural differences in unmet need for FP.

In this study, attending group FP counseling services with a healthcare provider reduced inequalities in unmet needs for FP between the rural and urban areas. This finding is consistent with another study conducted in Ethiopia, where visiting a healthcare provider and receiving counseling from a health professional reduces unmet needs for FP among reproductive-aged women [16, 17]. This may be because women who are counseled by a healthcare provider will more likely have the chance to get the information on where and how to access FP services [16]. In addition, the reduced gap between the urban and rural areas may be attributed to Ethiopia’s Health Extension Program (HEP), which predominantly focuses on the promotion of maternal and reproductive healthcare service utilization in the rural parts of Ethiopia, which may have resulted in increasing access to FP services among the rural women thereby reducing the gap with the urban areas in regard to healthcare services utilization [36].

In this study, material factors were the second largest contributor to urban-rural inequalities in unmet need for FP. Among these factors, the wealth index significantly contributed to urban-rural inequalities in the unmet need for FP among reproductive-aged women in Ethiopia. Being from the 1st and 2nd quintile (representing the poorest and poorer groups, respectively) contributed to an increase in the gap in unmet FP needs between urban and rural women. This finding is supported by a report from the Ethiopian Development Research Institute (EDRI), which showed that poverty is largely concentrated in the rural areas (80%) of Ethiopia and to a lesser extent in urban areas [37]. According to the EDRI report, this may be due to more advanced industrialization and infrastructures in urban areas, which are likely to increase people’s access to better health and education services and in turn lead to better jobs and income for urban households [37]. Other studies from Ethiopia, Burundi, and Pakistan have likewise associated lower household wealth with an unmet need for FP [13–15], which may be explained by increased health-seeking behavior among women with higher income or wealth status [15].

Although prior studies identified a significant association between low educational level and unmet need for FP [11, 13, 14], this study showed a non-significant contribution of educational level to the urban-rural inequalities in unmet need for FP. Future studies should assess to which degree other factors, in particular psychosocial factors, may mask the effect of education on unmet FP need inequalities.

Religion was the only cultural-behavioral factor that has a statistically significant contribution to urban-rural inequalities in unmet need for FP among reproductive-aged women in Ethiopia. According to this study, being a Muslim contributed to the gap in unmet need for FP between urban and rural residents. This finding can be supported by prior studies conducted in both Ethiopia and Malawi, which showed that Muslim women have a higher prevalence of unmet need for FP than Christian women [9, 10]. In addition, a study conducted in Nigeria have also shown that being Muslim increases urban-rural inequalities in postpartum FP use [38]. Various religious beliefs may have differential effects on an individual’s health behaviors. For example, a prior study from Iran showed that a strong religious belief is associated with higher fertility preferences, and this may adversely impact women’s FP use [39].

Being between the age of 15–24 and 25–34 years reduced urban-rural inequalities in unmet need for FP as compared to those aged 35–49 years. This may be due to the implementation of various community-based programs such as the Women’s Developmental Army program, which is more common in the rural and benefits rural women to be more knowledgeable and motivated to use FP services than their urban counterparts [40].

### Strengths and limitations of the study

This is the first study that disentangled and assessed the contributory factors for urban-rural inequalities in unmet need for FP in Ethiopia. It is based on nationally representative data with a high (98.5%) response rate. Since the PMA survey has been conducted in eight other countries in Africa and Asia, the present results may guide other studies to investigate and compare the contributing factors to urban-rural inequalities in FP needs in various contexts. In particular, certain study variables were pre-calculated by PMA (e.g., unmet need for FP, wealth index) which makes the present results directly comparable to other PMA studies.

However, some limitations should be considered while interpreting the results. First, because of the data limitations, the neo-material variables such as the implementation of reproductive health policies were not included, which could have affected the results. Second, since the contributory factors and the outcome were assessed at the same point of time, we may underestimate the true effect of the contributory factors. Third, the dichotomized outcome variable, unmet need for FP, does not allow to look at the severity or the gradient of unmet FP needs. Finally, because of the cross-sectional study design used in this study, causality cannot be established.

## Conclusion and recommendations

In conclusion, this study showed that among reproductive-aged women in Ethiopia, inequalities in unmet FP needs show distinct urban-rural patterning. Most inequalities could be attributed to psychosocial factors, mainly parity and marital status, followed by material factors such as household wealth and cultural-behavioral factors.

In order to reduce such inequalities, policymakers and higher officials should focus and act upon modifiable factors that contribute to urban-rural inequalities. To improve FP services and reduce unmet FP needs, Ethiopia has undertaken a number of initiatives, including increased human capital and the number of health facilities offering FP counseling and provision services. In addition to these efforts, policymakers and higher officials should increase various infrastructures and innovative strategies to improve household wealth among rural communities. Group FP counseling services with a healthcare provider should also be strengthened and widely disseminated in both rural and urban areas. Similarly, strategies that can increase multipara’s awareness regarding contraceptive use, as well as access to FP services, should be more widely implemented. Finally, our results also speak toward the benefits of involving higher religious leaders in creating FP awareness in larger faith communities.

## Data Availability

Data used for this study is available to the public from the PMA website (https://www.pmadata.org/countries/ethiopia).
